# Operational research capacity building using ‘The Union/MSF’ model: adapting as we go along

**DOI:** 10.1186/1756-0500-7-819

**Published:** 2014-11-19

**Authors:** Ajay MV Kumar, Rony Zachariah, Srinath Satyanarayana, Anthony J Reid, Rafael Van den Bergh, Katie Tayler-Smith, Mohammed Khogali, Anthony D Harries

**Affiliations:** International Union Against Tuberculosis and Lung Disease (The Union), South-East Asia Regional Office, New Delhi, 110016 India; Operational Research Unit (LuxOR), Médecins Sans Frontières, Operational Centre Brussels, MSF-Luxembourg, Luxembourg; International Union Against Tuberculosis and Lung Disease (The Union), Paris, France; London School of Hygiene and Tropical Medicine, Keppel Street, London, UK

**Keywords:** Operational research, Training, Asia, Capacity building, SORT IT

## Abstract

**Background:**

We have conducted 23 operational research (OR) courses since 2009, based on ‘The Union/ Médecins Sans Frontières (MSF)’ model, now popularly known as SORT-IT (Structured Operational Research and Training Initiative) model - wherein participants are mentored through the whole research process from protocol development (module 1) to data analysis (module 2) to publication (module 3) over a period of 9–12 months. We have faced a number of challenges including shortage of time, especially for data analysis and interpretation, and a heavy mentorship burden on limited numbers of experienced facilitators. To address these challenges, we have made several modifications to the structure of the OR course. In this article, we describe the revised structure and our experience (successes and challenges) of implementing it in Asia in 2013.

**Findings:**

The key changes introduced included extending the duration of the course modules (by a day each in module 1 and 2 and by three days in module 3), increasing the numbers of facilitators and standardizing milestones related to data entry and analysis. We successfully implemented this revised structure in the second Asian OR Course held in Nepal in 2013. Eleven of twelve participants successfully completed all the milestones and submitted 13 scientific manuscripts (two participants completed two projects) to international peer-reviewed journals. Though, this posed two challenges – increased costs and increased time away for faculty and participants.

**Conclusions:**

The revised structure of ‘The Union/MSF’ model of OR capacity building addressed previous issues of insufficient time and overburdened mentors and we intend to continue with this model for future courses.

## Background

Since 2009, the International Union Against Tuberculosis and Lung Disease (The Union) and Médecins Sans Frontières (MSF) have been involved in building capacity of health professionals in low- and middle-income countries to conduct and publish operational research (OR). We use a practical and output-based approach with hands-on mentorship and has been described in detail elsewhere [1–3]. In brief, this model is implemented over a period of 9–12 months and consists of three modules – Module 1 on ‘research protocol development’, Module 2 on ‘data entry and analysis’ and Module 3 on ‘scientific paper writing’. To be successful, participants have to design and conduct an OR project and at the end of the course submit a scientific manuscript to a peer-reviewed journal. We have achieved excellent results with this model, with more than 85% of participants completing the course and more than 80% of submitted manuscripts being published. The Special Programme for Research and Training in Tropical Diseases (TDR) at the World Health Organization (WHO) has adopted this model as “The Structured Operational Research and Training InitiaTive (SORT IT)” and is committed to its global expansion [3].

To date, we have conducted 23 courses (17 courses completed and six on-going) based on The Union/MSF model. Over the years, we have faced a number of challenges in implementation of these courses, including shortage of time for data analysis, data interpretation and manuscript drafting, and a high mentorship burden on a limited pool of facilitators [4]. To address these challenges, we have introduced several changes to the structure of the OR course. In this article, we summarize these changes and share our experience of implementing a revised OR course structure in Asia.

## Findings: Contents of the adapted course

The changes made to the course structure are summarized in the Table 1. They include an increase in the number of days for each module, an increase in the number of facilitators and strengthened milestones related to Module 2. In Module 1, an additional day was used to introduce new sessions on how to systematically search for published literature and organize references. In Module 2, an added day was dedicated entirely to data analysis. Module 3 was extended by three days in order to a) provide more intensive and tailored support to participants for analysing their data, b) give participants more time to conduct a thorough on-line literature review and c) include a new plenary session on manuscript ‘titles and abstracts’. The number of facilitators was increased and standardised for each module, in order to ensure similar standard mentor-participant pairings in Modules 1 and 3, and to allow junior facilitators the opportunity to be trained by senior facilitators within each pairing group. The milestones relating to Module 2 were strengthened and standardized (Table 1).

**Table 1:** **Comparison of the initial and revised structure of the Union-MSF model of operational research course**

Aspect	Initial model	Revised model
**Duration of the module**	Each module was five days in duration. In Module 2, about 3.5 days were used on data entry and the rest on data analysis. While some courses offered tailored support on data entry and data presentation in function of the participant OR projects, no focus could be placed on data analysis in function of the specific projects.	Duration of Module 1 and 2 increased to six days while that of Module 3 increased to eight days.
		In Module 1, the extra day was used to introduce two new sessions – one on the systematic search of published literature and another on organizing references.
		In Module 2, we allocated two days for data entry, two days for data analysis, one day to develop data entry tools and the data-analysis plan for the participants’ research projects, and one day for plenary for presenting the data entry formats and dummy analytic tables.
		In Module 3, the first two days (Friday and Saturday) were dedicated to data analysis and interpretation followed by a day’s break (Sunday) for self-study and reading published literature. Projects requiring ‘multivariate regression analyses’ were supported on a case-to-case basis. This was followed by five days (Monday to Friday) for drafting the manuscript. A new plenary was introduced for presenting ‘titles and abstracts’.
**Number of facilitators**	In previous courses, the number of facilitators for Modules 1 and 3 varied from 6 to 9 and the number of mentor groups varied from 3 to 4. The facilitators worked in pairs - one senior (relatively more experienced in conducting and publishing OR) and one junior facilitator (usually one of the successful participants in the previous courses).	Number of facilitators in Modules 1 and 3 was standardized to eight (each pair of facilitators with three mentees)
	For Module 2, facilitators varied in number from two to six and there were a variable number of participants per facilitator.	Number of facilitators in Module 2 was increased and standardized to six – each had two mentees
**Strengthened milestones**	The milestones attached to Module 2 were weak, subjective and relied upon a self-declaration by the participant prior to Module 3 that the data collection had been completed.	The milestones related to Module 2 were modified and made more objective – one to be met within two weeks of Module 2 (submission of a plan for data collection, electronic data capture formats in EpiData (http://www.epidata.dk) and dummy tables and figures to facilitate analysis and reporting). The second milestone at least six weeks prior to Module 3 included submission of proof of study completion including the dataset and a draft analysis.

We implemented this revised structure in the second Asian OR Course held in Nepal in 2013. We had twelve participants, mostly health professionals working in programmes from India, China, Nepal, Bhutan, Bangladesh, Pakistan and Sri Lanka. Eleven of twelve participants successfully completed all the milestones with two of them completing two research projects each. Thus a total of 13 scientific manuscripts were submitted to international peer-review journals. One participant was not able to complete the research project in time due to changing her place and institution of work. The key advantages and challenges of the revised structure, as mentioned in the end-of–module feedback of participants and facilitators are summarized in the Table 2.

**Table 2:** **Advantages and disadvantages of the revised structure of ‘The Union-MSF model’ in Asia, 2013**

Revised structure	Advantages	Disadvantages
Extended duration of module	• Allowed new knowledge on sourcing published literature and organizing references to be imparted to participants	• Increased costs due to additional accommodation, conferencing and per-diem expenses
	• More individualized time devoted to analysing and interpreting data	• Increased time away from duty station for faculty and participants
	• Improved manuscript titles and abstracts. Less stress and fewer hours worked beyond course schedule for both participants and faculty	
	• Improved opportunities for social networking and alumni links between participants and mentors	
Increased number of facilitators	• For Modules 1 and 3, facilitator numbers were standardized to two for three participants allowing more individual time per participant	• Increased costs
Strengthened milestones for Module 2	• Increased priority accorded to data entry and analysis	• Increased burden on the participants and the facilitators to meet milestones
	• Increased hands-on support to participants in analyzing data	• Increased burden on the module coordinator and course co-ordinator to monitor the achievement of milestones

## Discussion

There were two main problems in our courses - shortage of time for data analysis and interpretation and high mentorship load. The problem of time shortage for data analysis was addressed in several ways. First, the additional days in Module 2 and 3 were primarily used to provide tailored data analysis support for research projects. Second, the number of facilitators for Module 2 was increased to six so that each facilitator supported only two participants in data analysis. Third, the strengthened milestone focusing on data analysis just before Module 3 increased the priority level and attention accorded to data analysis by participants and their mentors.

To reduce the high mentorship load, the module duration was extended which took pressure off both facilitators and participants, who on previous courses often worked late into the night and way beyond the course timetable. In the revised structure, participants had sufficient time to develop the first drafts of their protocols, carry out the data analysis and draft their manuscript before facilitators provided their inputs, thus enhancing the iterative learning experience. The revised structure also demonstrated the increased emphasis on reviewing published literature and organizing references, which had a limited focus in previous courses. This led to increased familiarity with previously published literature and improvements to both the introduction and discussion sections of the final papers. With the perspective of decentralizing OR courses to settings with relatively inexperienced and junior facilitators, and with growing diversification of the research portfolio beyond the current focus of HIV and tuberculosis, additional days have proved necessary to ensure the quality of outputs.

The two key challenges in implementing the revised structure were the associated increased costs (primarily for hotel and per-diem on additional days) and faculty having to commit to being away from duty stations for a longer period of time. The increased costs need to be included in future funding proposals to donors. Faculty commitment will be an ongoing challenge but will be partly solved by developing and nurturing a pool of senior and junior facilitators. It will be important to encourage facilitators who attend Module 1 to also attend Module 3 so that there is continuity and familiarity of faculty with the OR protocols.

In conclusion, the revised structure of ‘The Union/MSF’ model of OR capacity building addressed previous issues of insufficient time and overburdened mentors and we intend to continue with this model for future courses. We will continue to evaluate the revised model of capacity building using the standard SORT-IT indicators [3].
